# Cancer-associated variants of human NQO1: impacts on inhibitor binding and cooperativity

**DOI:** 10.1042/BSR20191874

**Published:** 2019-09-06

**Authors:** Clare F. Megarity, David J. Timson

**Affiliations:** 1 School of Biological Sciences, Queen’s University Belfast, Medical Biology Centre, 97 Lisburn Road, Belfast BT9 7BL, U.K.; 2 School of Pharmacy and Biomolecular Sciences, University of Brighton, Huxley Building, Lewes Road, Brighton BN2 4GJ, U.K.

**Keywords:** cancer-associated polymorphism, dicoumarol, DT-diaphorase, NAD(P)H quinone oxidoreductase, negative cooperativity, resveratrol

## Abstract

Human NAD(P)H quinone oxidoreductase (DT-diaphorase, NQO1) exhibits negative cooperativity towards its potent inhibitor, dicoumarol. Here, we addressed the hypothesis that the effects of the two cancer-associated polymorphisms (p.R139W and p.P187S) may be partly mediated by their effects on inhibitor binding and negative cooperativity. Dicoumarol stabilized both variants and bound with much higher affinity for p.R139W than p.P187S. Both variants exhibited negative cooperativity towards dicoumarol; in both cases, the Hill coefficient (*h*) was approximately 0.5 and similar to that observed with the wild-type protein. NQO1 was also inhibited by resveratrol and by nicotinamide. Inhibition of NQO1 by resveratrol was approximately 10,000-fold less strong than that observed with the structurally similar enzyme, NRH quinine oxidoreductase 2 (NQO2). The enzyme exhibited non-cooperative behaviour towards nicotinamide, whereas resveratrol induced modest negative cooperativity (*h* = 0.85). Nicotinamide stabilized wild-type NQO1 and p.R139W towards thermal denaturation but had no detectable effect on p.P187S. Resveratrol destabilized the wild-type enzyme and both cancer-associated variants. Our data suggest that neither polymorphism exerts its effect by changing the enzyme’s ability to exhibit negative cooperativity towards inhibitors. However, it does demonstrate that resveratrol can inhibit NQO1 in addition to this compound’s well-documented effects on NQO2. The implications of these findings for molecular pathology are discussed.

## Introduction

Human NAD(P)H quinone oxidoreductase 1 (NQO1, DT-diaphorase, EC 1.6.5.2) is an oxidoreductase flavoenzyme [1–4]. It catalyses the two-electron reduction of quinone substrates through a substituted enzyme (or “ping-pong”) mechanism where the electron donor (NAD(P)H) enters the active site, reduces the FAD cofactor by donation of two hydride ions, leaves the active site in its oxidized form (NAD^+^) and is replaced by the quinone substrate, which is subsequently reduced [5–7]. NQO1 is a homodimer with interlocking subunits. There are two active sites located at the dimeric interface and residues from each monomer together with the FAD cofactor form the boundaries of the active sites [8–11].

The two-electron reduction catalysed by NQO1 ensures the reduction of quinones directly to hydroquinones thereby avoiding the production of semiquinones. Semiquinones can be oxidized by molecular oxygen, resulting in the formation of superoxide (O_2_^−•^) free radicals [1,12,13]. Additionally, NQO1 directly scavenges superoxide free radicals and plays a minor role in the redox cycling of vitamin K by detoxifying vitamin K3 to the corresponding hydroquinone [14–17]. NQO1 also detoxifies benzoquinones, toxins which are produced by the metabolism of benzene and, therefore, a deficiency in NQO1 leads to increased levels of benzene-induced toxicity [18–20]. NQO1 also has roles in cancer and some neurological diseases [4]. Inhibition of the proteasome is involved in the pathogenesis of Parkinson’s disease and NQO1 helps to protect against this inhibition by reducing the endogenous proteasome inhibitor, aminochrome, to a cyclized hydroquinone [21,22]. NQO1 has also been implicated in the regulation of oxidative stress associated with Alzheimer’s disease [23–27].

In addition to protection afforded by its catalysis, NQO1 offers further chemoprotection by binding to, and stabilizing, the tumour-suppressing proteins, p53 and p73 and also the polyamine biosynthesis pathway enzyme, ornithine decarboxylase [28–32]. In its reduced state, NQO1 regulates the ubiquitin-independent proteasomal degradation of the tumour-suppressor proteins by association with the 20S proteasome and with p53 and p73 [31]. NQO1 also stabilizes the short-lived component of the transcription factor AP-1, c-fos. It stabilizes newly synthesised c-fos until it complexes with c-jun to form AP-1, which in turn translocates to the nucleus [33]. The transcription factor, 4GI, is also stabilized by NQO1 [34].

Additionally, the reduction catalysed by NQO1 has been exploited in the design of anti-cancer prodrugs. Generally, the reduction of quinones to hydroquinones is detoxifying because the donation of two electrons to the quinone ensures that highly reactive semiquinones are not produced. However, there are examples of quinones (natural and synthetic) whose chemical reactivity leads to the production of cytotoxic hydroquinones, for example, synthetic quinones can be designed so that, on reduction, they lose a leaving group to yield a hydroquinone which is a reactive electrophile which can alkylate DNA [35]. Such quinones can be exploited as anti-cancer prodrugs which become active when reduced by NQO1 and its high expression in tumour cells ensures that the effects of such prodrugs is largely targeted to these cells [35–37].

NQO1 is inhibited competitively by the coumarin-derivative, dicoumarol (an anticoagulant drug) which binds in the active site [5]. It π-stacks with the isoalloxazine ring of the FAD and forms hydrophobic interactions and hydrogen bonds with residues from both subunits [11]. Dicoumarol induces negative cooperativity in NQO1 [38–40]. Negative cooperativity is a decrease in affinity when sequential molecules of ligand bind. A redistribution of the native state ensemble of conformers occurs on binding of the first ligand molecule; in the new ensemble, the geometry of the second binding site is changed [41,42]. A conformational change which occurs on binding of the first molecule is propagated through the enzyme and causes the change in geometry at the second binding site. This requires a pathway linking the two active sites and in the case of NQO1, which has been investigated by molecular dynamics and site-directed mutagenesis experiments [39]. Glycine 150 has been identified as pivotal to information transmission since alteration to the more conformationally restricting serine largely abolished negative cooperativity towards dicoumarol [39].

Two polymorphic forms of human NQO1 have been associated with increased cancer risk: the NQO1*2 allele (rs1800566) and the NQO1*3 allele (rs1131341). These encode a proline to serine substitution at position 187 (p.P187S) and an arginine to tryptophan substitution at position 139 (p.R139W), respectively [43–45]. Arginine 139 forms part of a solvent-exposed loop located in the larger, N-terminal domain of the enzyme. Proline 187 is also located in the N-terminal domain and is part of a loop close to the surface of the protein [8]. Neither position occurs within the pathway involved in communication between the active sites that results in negative cooperativity towards dicoumarol [39]. Biochemical and biophysical studies have established that p.P187S has a much reduced affinity for FAD when compared with the wild-type protein [32,46–50]. Both cancer-associated variants have reduced overall stability compared with wild-type and p.P187S has been shown to be partially unfolded [46,47,51,52].

Here, we studied the effects of the two cancer-associated polymorphisms on dicoumarol binding and its inhibition, to address the hypothesis that the cancer-associated variants might respond differently to these inhibitors, particularly in regards to their negative cooperativity. Other potential inhibitors, resveratrol and nicotinamide, were also investigated in terms of binding, inhibition of oxidoreductase activity and ability to induce cooperativity in NQO1.

## Materials and methods

### NQO1 expression, purification and enzyme kinetics analysis

Hexahistidine-tagged human wild-type NQO1 and the two cancer-associated variants (p.R139W and p.P187S) were expressed in, and purified from, *Escherichia coli* as previously described [47]. Rates of reaction were measured using NADH or NADPH as a reducing agent and DCPIP as the second substrate [47]. Briefly, rates were measured at 37°C in Hepes-OH buffer (pH 7.3) and were obtained from the linear section at the beginning of each progress curve. Each inhibitor was added into the reaction (dicoumarol: 0–20 nM for wild-type and p.R139W and 0-50 nM for p.P187S; resveratrol: 0–500 μM; nicotinamide: 0–80 mM) and the effect on the enzyme-catalysed rate measured at, at least two concentrations of NAD(P)H and one DCPIP concentration (70 μM). Dicoumarol was dissolved in 0.13 M NaOH and the final concentration of NaOH was in all reactions (including those with zero inhibitor) was 0.65 mM. Resveratrol was dissolved in DMSO and the final volume of DMSO in each reaction was 0.5% v/v including reactions for zero inhibitor. Nicotinamide was dissolved in the buffer used for the reaction. Dixon plots (1/*v* against [Inhibitor]) were constructed and the apparent inhibition constant, *K*_i,app_ calculated [53]. Plots of [*S*]/*v* were also constructed for each concentration of NAD(P)H and, together with the corresponding Dixon plot, were used to determine whether the inhibition was strictly competitive or whether it was mixed [54]. A range of inhibitor concentrations were used (in triplicate of triplicate) to construct a Hill plot using 70 μM DCPIP and 300 μM NADH. Linearized Hill plots of (−log_10_(*v*/(*v*_0 −_ *v*))) against (-log_10_[Inhibitor]) (where *v* is the rate and *v*_0_ is the rate in the absence of inhibitor) were plotted for each inhibitor, the gradient of this linearized plot is the Hill coefficient, *h* [55–57]. Experiments to investigate inhibition by dicoumarol were repeated in the presence of excess FAD. For these reactions, enzyme was pre-diluted using a buffered solution of FAD such that the concentration of FAD was 10 times the concentration of active sites in the diluted stock.

### Differential scanning fluorimetry

Differential scanning fluorimetry (DSF) was carried out using a Rotor-Gene Q cycler (Qiagen). The natural fluorescence resulting from the release of FAD on thermal denaturation was exploited as previously described [39,58–60]. An initial experiment ([wild-type NQO1] and [p.R139W] = 0.25–5 μM dimer; [p.P187S] = 0.25–20 μM dimer) identified an enzyme concentration which gave an optimal fluorescent signal. The apparent binding constants were determined by plotting the change in melting temperature (Δ*T*_m_) of each variant at each concentration of inhibitor against the corresponding inhibitor concentration. The data were fitted to (eqn 1) using non-linear curve fitting in GraphPad Prism 6.0 (GraphPad Software Inc., CA, U.S.A.).

(1) $$\Delta T_{m}=\frac{\left(\Delta {\text{T}}_{m,\max}[\text{Inhibitor}]\right)}{\left({\text{K}}_{D,\mathrm{app}}+\left[\text{Inhibitor}\right]\right)}$$

Where Δ*T*_m_,_max_ is the maximum, limiting change in *T*_m_ and *K*_D_,_app_ is the apparent dissociation constant for the inhibitor and protein.

## Results

### Both cancer-associated variants show negative cooperativity towards some inhibitors

**Figure 1:**
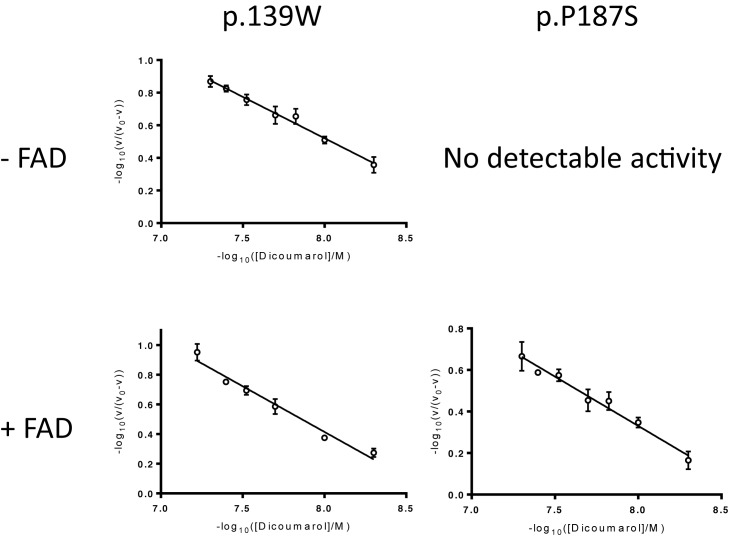
Inhibition of cancer-associated variants of human NQO1 by dicoumarol Top row: linear Hill plots constructed using inhibition data in the absence of additional FAD. Rates were measured HEPES-OH buffer pH 7.3 at 37°C in the presence of 0.9 μM lysozyme (as a crowding agent) with 70 μM DCPIP and 300 μM NADH; [p.R139W] = 1nM dimer. Bottom row: Hill plots constructed using inhibition data in the presence of excess FAD (10x [active sites]); [p.R139W] = 1.0 nM dimer; [p.P187S] = 20 nM dimer. Each point represents the mean of three separate determinations and the error bars show the standard error of these means. One representative Hill plot is shown from a triplicate set.

**Figure 2:**
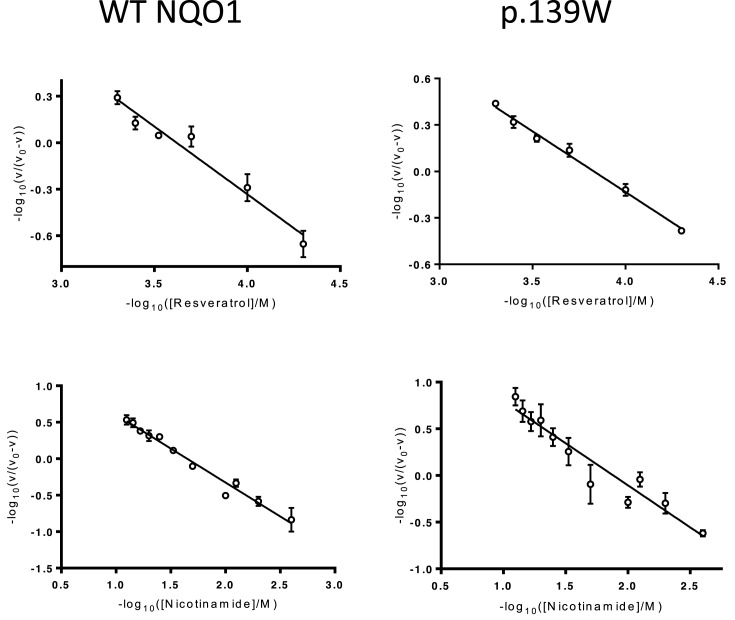
Inhibition of wild-type NQO1 and p.R139W by resveratrol Linear Hill plots constructed using resveratrol and nicotinamide inhibition data. Rates were measured in HEPES-OH buffer, pH 7.3, at 37°C in the presence of 0.9 μM lysozyme with 70 μM DCPIP and 300 μM NADH. [WT-NQO1] = 1.0 nM dimer; [p.R139W] = 1.0 nM dimer. Each point represents the mean of three separate determinations and the error bars the standard error of these means. One representative Hill plot is shown from a triplicate set.

**Figure 3:**
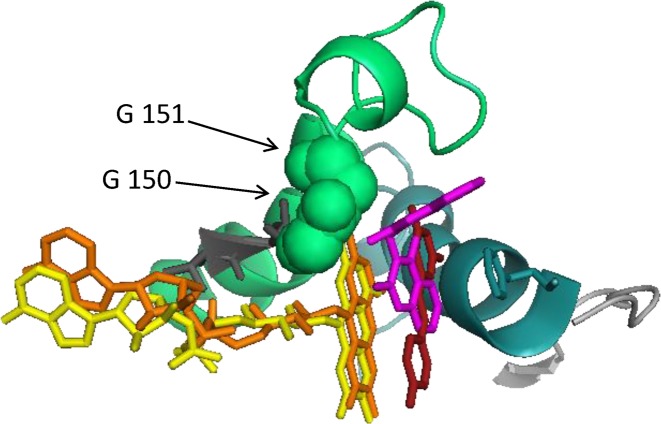
Structural alignment of NQO1 with NQO2 Dicoumarol is shown in pink, resveratrol in red, FAD from NQO2 in orange and FAD from NQO1 in yellow. Resveratol is flat and does not make contact with the glycine residues at position 150 and whereas dicoumarol is bent and does make contact with these residues. The alignment was made using PyMol (www.pymol.org), the command used aligns all atoms but includes an outlier rejection to ignore parts that deviate by more than 2 Å. Structures were taken from: NQO1, PDB 2F1O [11] with FAD from PDB 1QBG [9] and resveratrol from PDB 1SG0 [61].

Both cancer-associated variants are competitively inhibited by dicoumarol with *K*_i,app_ values in the nM range (Table 1). Interestingly, p.P187S is inhibited less by dicoumarol (*K*_i,app_ value approximately ten times higher than wild-type). This most likely reflects the lower thermal stability of this variant: a greater fraction of the protein molecules are likely to be partly unfolded and only properly folded protein is likely to be able to bind to the inhibitor. This inhibition exhibited negative cooperativity towards dicoumarol with Hill coefficients close to 0.5 for all three forms of the protein (Figure 1 and Table 1). Dicoumarol-induced negative cooperativity also occurred when the experiment was repeated after premixing the variants with excess FAD (Figure 1 and Table 1). This ruled out the possibility that the results were due to the different FAD content of the variants. Resveratrol also inhibited the oxidreductase activity of NQO1. The strength of inhibition (as estimated from the *K*_i,app_ values determined under similar conditions) is approximately 10,000-fold less than that observed for the structurally related enzyme NRH quinine oxidoreductase 2 (NQO2; EC 1.10.5.1) (Table 1) [58]. In contrast with dicoumarol, resveratrol’s inhibition of NQO1 was not wholly competitive. Dixon plots and corresponding [*S*]/*v* plots for the wild-type enzyme indicate that the inhibition was competitive with respect to the electron donor, NADPH but mixed with respect to NADH (data not shown). Resveratrol inhibition of the p.R139W variant was mixed with respect to NADPH and uncompetitive with respect to NADH (data not shown). The wild-type enzyme and p.R139W exhibited slight negative cooperativity towards resveratrol, although not to the same extent as dicoumarol, with Hill coefficients close to 0.85 (Table 1 and Figure 2). Moreover, based on structural alignment with human NQO2 in complex with resveratrol (PDB: 1SG0) [61], the probable binding orientation of resveratrol to human NQO1 suggests that it is unlikely to bind in close contact to the glycine residues at positions 149 and 150 since it is flat and not hinged like dicoumarol (Figure 3); this may explain why, unlike dicoumarol, it only induces slight negative cooperativity.

**Table 1:** Inhibition constants (*K*_i,app_), Hill coefficients (*h*) and apparent dissociation constants derived from DSF experiments (*K*_D,app_) for wild-type NQO1 and the variants p.R139W and p.P187S

Inhibitor	Wild-type NQO1	p.R139W	p.P187S
	***K***_i,app_		
Dicoumarol	1.15 ± 0.51 nM	2.98 ± 1.04 nM	10.95 ± 1.11 nM^2^
Nicotinamide	14.01 ± 4.34 mM	13.55 ± 3.22 mM	nd
Resveratrol	375 ±129 μM	nd	nd
	**Hill coefficient (*h*)**		
Dicoumarol	0.56 ± 0.09	0.51 ± 0.09	Inactive
Dicoumarol + excess FAD	0.47 ± 0.09^1^,^2^	0.60 ± 0.04^2^	0.48 ± 0.02^2^
Resveratrol	0.89 ± 0.08	0.85 ± 0.12	nd
Nicotinamide	0.91 ± 0.06	0.92 ± 0.05	nd
	***K*_D,app_**		
Dicoumarol	54 ± 4 nM	66 ± 11 nM	1100 ± 280 nM
Nicotinamide	6.0 ± 0.9 mM	18.2 ± 5.2 mM	nd

*h* is the mean value from three separate linear Hill plots ± the standard deviation of this mean.

1 Previously reported and included here for comparison [39].

2 In excess FAD (10×[active site]).

nd, not determined.

Nicotinamide inhibited NQO1 and the type of inhibition was mixed. Wild-type NQO1 and the p.R139W variant did not exhibit cooperativity towards nicotinamide (Table 1 and Figure 2). This is expected since nicotinamide is a building block of the electron donors, NADH and NADPH, neither of which induce cooperativity [38,39]. This may be because the nicotinamide moiety does not make the required contacts with glycines at position 149 and 150 to induce cooperativity [10].

### Inhibitor binding affects the stability of wild-type and variant NQO1

**Figure 4:**
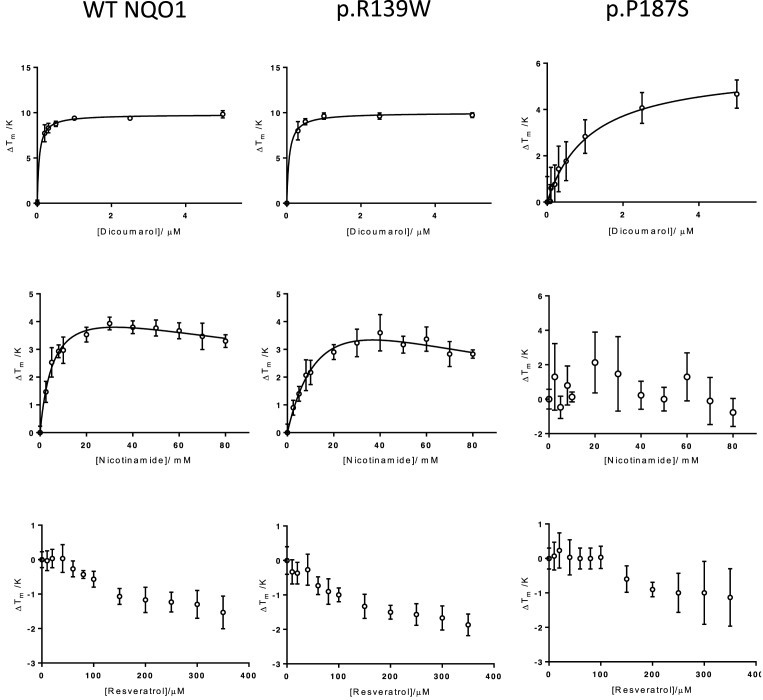
Thermal stability of wild-type human NQO1 and its cancer-associated variants The change in melting temperature (Δ*T*_m_) was plotted against the corresponding inhibitor concentration. Each point represents the mean of three values and the error bars the standard error of these means. Top row: stabilization by dicoumarol. Triplicate samples of wild-type NQO1, p.R139W and p.P187S (2, 2 and 15 μM dimer, respectively) in HEPES-OH buffer (pH 7.3) mixed with increasing concentrations of dicoumarol (in 0.13 M NaOH; final [NaOH] constant in each sample (0.65 mM). Middle row: stabilization by nicotinamide. Triplicate samples of wild-type NQO1, p.R139W and p.P187S (2, 2 and 15 μM dimer, respectively) in HEPES-OH buffer (pH 7.3) mixed with increasing concentrations of nicotinamide. Bottom row: destabilization by resveratrol Triplicate samples of wild-type NQO1, p.R139W and p.P187S (2, 2 and15 μM dimer, respectively) in HEPES-OH buffer (pH 7.3) mixed with increasing concentrations of resveratrol (resveratrol dissolved in DMSO; [DMSO] constant in all reactions at 0.5% (v/v)).

Dicoumarol bound to and stabilized each variant towards thermal denaturation in a concentration-dependent manner (Figure 4). The *K*_D,app_ values for dicoumarol determined by differential scanning fluorimetry are unlikely to reflect the true equilibrium constants for the inhibitor–protein interaction. Indeed, they are several order of magnitude different from the apparent inhibition contains derived from kinetic analyses (Table 1). However, there is good reason to believe that they do enable the ranking of affinities and the trend follows that of the apparent inhibition constants. Both sets of values indicate comparably tight binding to the wild-type enzyme and the p.R139W variant and less tight binding to the p.P187S variant (Table 1). This is consistent with previous studies using isothermal titration calorimetry [49].

Nicotinamide stabilized the wild-type enzyme and p.R139W towards thermal denaturation in a concentration-dependent manner but had no detectable effect on p.P187S (Figure 4). Since wild-type and p.R139W are active and folded [47], nicotinamide’s stabilization of these variants indicates that it binds preferentially to the folded state [62]. This may also explain its inability to stabilise p.P187S: the entropic cost to this variant on binding ligand may be greater than that for wild-type or p.R139W and the enthalpically favourable contacts which would occur on binding nicotinamide may not outweigh the entropic cost for p.P187S.

The effects of resveratrol on thermal stability were less clear. This compound destabilised wild-type NQO1 and both cancer-associated variants (Figure 4). While the effect was clearly concentration dependent, it could not be fitted to any simple model (data not shown). This is in contrast with the related human enzyme, NQO2, which is stabilised by resveratrol [58]. Destabilization by a ligand can indicate that the ligand binds more favourably to partially unfolded molecules [62].

## Discussion

The physiological significance of the negative cooperativity towards inhibitors in NQO1 is not known. It has been postulated that it may be involved in regulating NQO1’s cellular activity by an as yet unidentified inhibitor or that it may be important in modulating the protein’s interactions with molecules such as p53 [39]. It may also partly reflect the recently documented negative cooperativity towards the cofactor, FAD since dicoumarol binding requires contacts with FAD as well as the protein itself [11,49,63].

However, the data presented here demonstrate that the cancer-associated polymorphisms in NQO1 do not affect the enzyme’s ability to exhibit negative cooperativity towards the inhibitor dicoumarol. This implies that neither polymorphism exerts its pathological effect by alteration of the enzyme’s negative cooperativity towards inhibitors. Since changing the residues at positions 139 and 187 had no effect on cooperativity, it can be concluded that arginine and proline at these respective positions are not involved in the mechanism of communication between each active site. This is consistent with previous work: these residues are not located within, or close to, residues predicted to be involved in the communication between active sites [39].

Inhibition of NQO1 has been suggested as a cancer therapy [64,65]. Dicoumarol inhibits the growth of some cancer cell lines, (e.g. pancreatic cancer cells [66]). This compound caused an increase in superoxide production (resulting from the one-electron reduction of quinones by cytochrome p450 reductase [66]) as a consequence of its inhibition of NQO1 and its activity as a mitochondrial uncoupling agent [67]. Thymoquinone (TQ) causes the production of superoxide radicals which induce apoptosis in breast cancer cells. Inhibition of NQO1 prevents it from scavenging these radicals [68]. The stabilization of the p.P187S variant by pharmacological chaperones has been suggested as a therapy for patients who are homozygous for the corresponding mutation [46,69]. Such reagents would restore FAD binding activity of this variant and, thus, its activity.

We have shown, for the first time, that resveratrol binds to NQO1 destabilizing it and inhibiting its oxidoreductase activity. The mixed nature of this inhibition may reflect a combination of two effects: competition for the substrate in the active site and reduction in protein stability. This inhibition by resveratrol could be exploited in the search for novel inhibitors of NQO1. However, the inhibition of NQO1 is much weaker than that of NQO2 (approximately 10^5^-fold) and so its physiological significance may be less important. Nevertheless, this effect should be considered in *in vitro* experiments. Where high (μM to mM) concentrations of resveratrol are used, it cannot be assumed that all effects are due to the inhibition of NQO2. At these concentrations, there will also be some effect on NQO1.

Taken together, our data demonstrate that modulation of negative cooperativity is unlikely to be involved in the molecular pathology of these cancer-associated polymorphisms. However, the reduction in dicoumarol affinity may be important. These suggest that other inhibitors based on dicoumarol may be less effective in patients with these polymorphisms (especially p.P187S). Furthermore, the lower thermal stability of the two variants reported here (and elsewhere [44,47,51,70]) will influence NQO1’s ability to interact with, and regulate, molecules such as p53, p73 and the 20S proteasome. It seems likely that this is the most important, underlying, biochemical reason why these variants lead to increased cancer risk.

## Abbreviations

DSF: differential scanning fluorimetry
NQO1: NAD(P)H quinone oxidoreductase 1
TQ: thymoquinone
